# Hypertension and hand-foot skin reactions related to VEGFR2 genotype and improved clinical outcome following bevacizumab and sorafenib

**DOI:** 10.1186/1756-9966-29-95

**Published:** 2010-07-14

**Authors:** Lokesh Jain, Tristan M Sissung, Romano Danesi, Elise C Kohn, William L Dahut, Shivaani Kummar, David Venzon, David Liewehr, Bevin C English, Caitlin E Baum, Robert Yarchoan, Giuseppe Giaccone, Jürgen Venitz, Douglas K Price, William D Figg

**Affiliations:** 1 Clinical Pharmacology Program, Center for Cancer Research, National Cancer Institute, (9000 Rockville Pike), Bethesda, (20892), USA; 2 Department of Pharmaceutics, School of Pharmacy, Virginia Commonwealth University, (410 N 12th Street, Richmond, 23298, USA; 3 Medical Oncology Branch, Center for Cancer Research, National Cancer Institute, (9000 Rockville Pike), Bethesda, (20892), USA; 4 Department of Internal Medicine, University of Pisa, (55 Via Roma), Pisa, (56126), Italy; 5 Biostatistics and Data Management Section, National Cancer Institute, (6116 Executive Boulevard), Bethesda, (20852), USA; 6 Molecular Pharmacology Section, Center for Cancer Research, National Cancer Institute, (9000 Rockville Pike), Bethesda, (20892), USA; 7 HIV and AIDS Malignancy Branch, National Cancer Institute, (9000 Rockville Pike), Bethesda, (20892), USA

## Abstract

**Background:**

Hypertension (HT) and hand-foot skin reactions (HFSR) may be related to the activity of bevacizumab and sorafenib. We hypothesized that these toxicities would correspond to favorable outcome in these drugs, that HT and HFSR would coincide, and that *VEGFR2 *genotypic variation would be related to toxicity and clinical outcomes.

**Methods:**

Toxicities (≥ grade 2 HT or HFSR), progression-free survival (PFS), and overall survival (OS) following treatment initiation were evaluated. Toxicity incidence and VEGFR2 H472Q and V297I status were compared to clinical outcomes.

**Results:**

Individuals experiencing HT had longer PFS following bevacizumab therapy than those without this toxicity in trials utilizing bevacizumab in patients with prostate cancer (31.5 vs 14.9 months, *n *= 60, *P *= 0.0009), and bevacizumab and sorafenib in patients with solid tumors (11.9 vs. 3.7 months, *n *= 27, *P *= 0.052). HT was also linked to a > 5-fold OS benefit after sorafenib and bevacizumab cotherapy (5.7 versus 29.0 months, *P *= 0.0068). HFSR was a marker for prolonged PFS during sorafenib therapy (6.1 versus 3.7 months respectively, *n *= 113, *P *= 0.0003). HT was a risk factor for HFSR in patients treated with bevacizumab and/or sorafenib (OR(95%CI) = 3.2(1.5-6.8), *P *= 0.0024). Carriers of variant alleles at VEGFR2 H472Q experienced greater risk of developing HT (OR(95%CI) = 2.3(1.2 - 4.6), *n *= 170, *P *= 0.0154) and HFSR (OR(95%CI) = 2.7(1.3 - 5.6), *n *= 170, *P *= 0.0136).

**Conclusions:**

This study suggests that HT and HFSR may be markers for favorable clinical outcome, HT development may be a marker for HFSR, and *VEGFR2 *alleles may be related to the development of toxicities during therapy with bevacizumab and/or sorafenib.

## Background

The process of angiogenesis is crucial for carcinogenesis, invasiveness and metastasis in several tumor types including prostate, ovary, kidney, non-small cell lung and colorectal cancer [1-3]. This process is governed by an array of growth factors; however, vascular endothelial growth factor (VEGF) and its major receptor in the endothelium, VEGFR2, are predominant regulators of this process [2]. Rising interest in angiogenic modulators has led to the design and synthesis of several new molecules that target the VEGF signaling pathway, such as sorafenib, bevacizumab and sunitinib, which are currently approved for various solid tumors. There is wide inter-individual variation in toxicity and clinical outcome following treatment with agents targeted at the VEGF pathway suggesting that predictive markers of these outcomes could be clinically useful.

Sorafenib and bevacizumab have some common toxicities, such as hypertension (HT), diarrhea, and gastrointestinal perforation [4,5]. However, sorafenib confers frequent cutaneous side effects, including hand-foot skin reaction (HFSR; palmar-plantar dysesthesia; acral erythema) and rash in many individuals while bevacizumab confers HFSR in a limited number of individuals. Both *in-vitro *and *in-vivo *evidence support that HT, results directly from the pharmacologic activity of VEGF inhibitors [6]. Recently, we demonstrated that sorafenib-induced HFSR was directly related to cumulative sorafenib dose, that HT and HFSR development coincided, and that HFSR is more prevalent in individuals being treated with a combination of sorafenib and bevacizumab targeting the VEGF receptor and the VEGF growth factor, respectively. A pharmacokinetic interaction was not observed [7]. Taken together, these results suggest that HFSR and HT may both be related to the activity of anti-VEGF and anti-VEGFR therapy; thus, HT and HFSR may also be markers for a greater degree of response in patients treated with sorafenib and bevacizumab. Inter-individual genetic variation in the VEGF pathway may also alter both the toxicity and response to these agents.

The *VEGFR2 *gene contains two SNPs that are located in exons 7 and 11 and result in nonsynonymous amino acid changes at residues 297 Val>Ile and 472 His>Gln in the third and fifth immunoglobulin like (Ig-like) domains of VEGFR2 receptor, respectively. The Ig-like domain 3 is critical for binding to the VEGF ligand [8], while domains 4-7 contain structural features that inhibit VEGFR2 signaling in the absence of VEGF [9]. HEK293 s cells that were transfected with VEGFR2 V297I SNP had significantly low VEGF binding efficiency regardless of VEGFR2 H472Q genotype, while variant VEGFR2 H472Q allele had minimal effect on VEGF binding efficiency [10].

We hypothesize that 1) the development of HT and HFSR following anti-VEGF therapy with bevacizumab and sorafenib is a marker for response to these drugs; 2) that since both toxicities are related to the activity of these agents, the development of a single toxicity (i.e. HT) would increase the risk of developing the other toxicity (i.e. HFSR); and 3) that functional SNPs in *VEGFR2 *could alter antiangiogenesis treatment response or outcome by affecting the VEGF signalling pathways. To this end, we determined if HT and HFSR were associated with progression free survival or overall survival, and if development of HT increased the risk of developing HFSR in patients with various solid tumors being treated with sorafenib and/or bevacizumab. We also determined if genetic polymorphisms in the *VEGFR2 *gene modified the relationship between toxicity and survival endpoints as well as the relationship between coincidence of HT and HFSR.

## Methods

### Patients and treatment

The analyses were performed on genomic DNA from 178 patients (143 males and 35 females) with solid tumors who received sorafenib (VEGFR2 inhibitor) and/or bevacizumab (anti-VEGF) with or without other agents. These patients were enrolled in six phase I or II clinical trials at the National Cancer Institute (Table 1). Two phase II trials (BAY-CRPC and APC-CRPC; NCT00093431 and NCT00091364 respectively on clinicaltrials.gov) in patients with castrate resistant prostate cancer (CRPC) administered sorafenib 400 mg bid and a combination of thalidomide (200 mg qhs), bevacizumab and docetaxel (15 mg/kg plus 75 mg/m^2 ^day 1, q 21 days), respectively [11,12]. Two other phase II trials (BAY-NSCLC and BAY-CRC; NCT00100763 and NCT00343772 respectively) treated patients with non-small cell lung cancer (NSCLC) [13] and colorectal cancer (CRC), respectively with sorafenib 400 mg bid and a combination of sorafenib (400 mg bid) and cetuximab (400 mg/m^2 ^loading dose in week 1 + 250 mg/m^2 ^i.v. week). Two phase I trials (BAY-BEV and BAY-KS; NCT00098592 and NCT00304122 respectively) administered sorafenib plus bevacizumab (200 mg bid + 5 mg/m^2 ^i.v. q15 days) [14] and sorafenib with or without a protease inhibitor (starting dose of 200 mg qd/bid ± starting dose of 200 mg qd) respectively to patients with solid tumors and Kaposi's sarcoma.

**Table 1:** Summary of patients included in analysis

Trial	Tumor type	Treatment (s)	*n*	Frequency of Toxicity [*n *= (%)]		Median PFS (months)			
			
				HT ≥ grade 2	HFSR ≥ grade 2	HT < grade 2 vs. ≥ grade 2	Log-Rank *P *=	HFSR < grade 2 vs. ≥ grade 2	Log-Rank *P *=
APC-CRPC	mCRPC	Bevacizuamb + Thalidomide + Docetaxel	60	15 (25.0)	4 (6.7)	14.9 vs. 31.5	**0.0009**	N/A*	ND*
BAY-BEV	ST	Sorafenib + Bevacizumab	27	15 (55.6)	13 (48.1)	3.7 vs. 11.9	0.052	3.7 vs. 12.6	0.094
BAY-CRPC^†^	mCRPC	Sorafenib	46	9 (19.6)	7 (15.2)	3.7 vs. 1.8	0.067	2.0 vs. 3.1	0.29
BAY-NSCLC	NSCLC	Sorafenib	22	9 (40.9)	10 (45.5)	1.9 vs. 4.6	0.19	2.9 vs. 3.7	0.38
BAY-CRC	CRC	Sorafenib + Cetuximab	18	1 (5.6)	2 (11.1)	N/A*	ND*	4.7 vs. 8.7	**0.0065**
BAY-KS^‡^	KS	Sorafenib +/- Protease inhibitor	8	3 (37.5)	2 (25.0)	N/A*	ND*	N/A*	ND*

*Not done (ND). Patients were not evaluated in this analysis due to low frequency of toxicity (i.e. APC-CRPC vs. HFSR and BAY-CRC vs. HT) or due to limited PFS data (KS).

^†^3 Patients participating on this trial were also treated on APC-CRPC.

^‡^Two patients on BAY-KS trial received only sorafenib.

**C**: Caucasian, **AA**: African-American, **Others**: Hispanic or Asians, **mCRPC**: metastatic castrate resistant prostate cancer, **NSCLC**: non-small cell lung cancer, **CRC**: colorectal cancer, **KS**: Kaposi's sarcoma, **ST**: solid tumors, **HFSR**: hand-foot skin reaction syndrome, **NA**: not applicable

The most severe grades of common, sorafenib treatment associated toxicities, namely rash, desquamation, diarrhea, HFSR, HT and fatigue were used for analysis. Toxicities were graded based on the National Cancer Institute common toxicity criteria version 3.0. This retrospective genotyping analysis was approved by the National Cancer Institute Institutional Review Board.

### Genotyping

DNA was extracted from plasma or whole blood using QiaBlood extraction kit (Qiagen, Valencia, CA). Genotyping for two *VEGFR2 *loci was performed by single/nested PCR using the following primers at an annealing temperature of 60°C: rs1870377 (T/A) F1:5'-CAGAATCACCCTACACAGATGC-3', R1: 5'-TTCCCAGAATAGCTGCTTCC-3', F2: 5'-TGGTACTGCTAAAAGTCAATGG-3', R2:5'-GGCTGCGTTGGAAGTTATTT-3'; and rs2305948 (C/T) F4: 5'-GGTTTGAACCCAAGTTCCTG-3', R4: 5'-CACTTTCACCACGTGAGGTTT-3', F5: 5'-TGGCCTCCCTAACAAGAAAA-3', R5: 5'-TGGTGTCCCTGTTTTTAGCA-3'. The details of the genotyping procedure are described elsewhere [15]. The sequencing PCR was carried out with Big Dye (v3.1, Applied Biosystems, Foster City, CA) using the following PCR primers: rs1870377 (T/A) F3: 5'-CCTGGAAGTCCTCCACACTT-3', R3: 5'-AACCAAAGTCTGAATCTTTTCCTT-3'; and rs2305948 (C/T) F6: 5'-CCCTGACAAATGTGCTGTTC-3', R6: 5'-TGCTGTGCTTTGGAAGTTCA-3'. The PCR products were then sequenced on an ABI Prism 3130xl Genetic Analyzer (Applied Biosystems) as per the instructions from the manufacturer.

### Statistical considerations

The progression free or overall survival based on genotype or toxicity groups (grade ≥ 2/grade < 2) was estimated by the Kaplan-Meier method [16] and compared by the exact log-rank test. Deviation from Hardy-Weinberg equilibrium was tested separately for different ethnic groups, using the Chi-squared test. The impact of genotypes on treatment-associated toxicities and the association between toxicities were assessed by Fisher's exact test. All statistical analyses were two-tailed at a pre-specified significance level of < 0.05. In view of the exploratory nature of analysis, *P*-values were not formally corrected for multiple testing. SAS for Windows version 9.1.3 was used for these statistical analyses.

## Results

### Genotyping data

The genotype and allele frequencies of studied *VEGFR2 *SNPs are shown in Table 2. Both *VEGFR2 *SNPs were in Hardy-Weinberg equilibrium (*P *≥ 0.77) when evaluated in Caucasian patients (*n *= 140) and African American patients (*n *= 17). Hardy-Weinberg equilibrium was not assessed in Hispanics and Asians (*n *= 13). There was no linkage between the two *VEGFR2 *SNPs (*P *> 0.05) in any of the studied populations.

**Table 2:** Genotype and allele frequencies for SNP in *VEGFR2 *loci for patients treated with sorafenib and/or bevacizumab, with or without other agents

Allelic variant	N	Genotype frequencies, N (%)			Allelic frequencies	
		Wt	Het	Var	p	q
**VEGFR2 H472Q**	170					

C*	140	82	50	8	0.76	0.24
AA*	17	12	5	0	0.85	0.15
**Others**	13	9	4	0	N/A	N/A

**VEGFR2 V297I**	170					

C*	140	114	25	1	0.9	0.1
AA*	17	9	6	2	0.71	0.29
**Others**	13	8	5	0	N/A	N/A

* Genotyping information was not available for *n *= 7 Caucasians and *n *= 1 African American included in subsequent analyses.

**C**: Caucasians, **AA**: African-Americans, **Others**: Hispanic or Asians, **Wt**: wild-type genotype, **Het**: heterozygous genotype, **Var**: homozygous variant genotype, **p and q **are standard Hardy-Weinberg nomenclature for allele frequencies.

### HT and HFSR as phenotypic markers for PFS and OS

**Figure 1:**
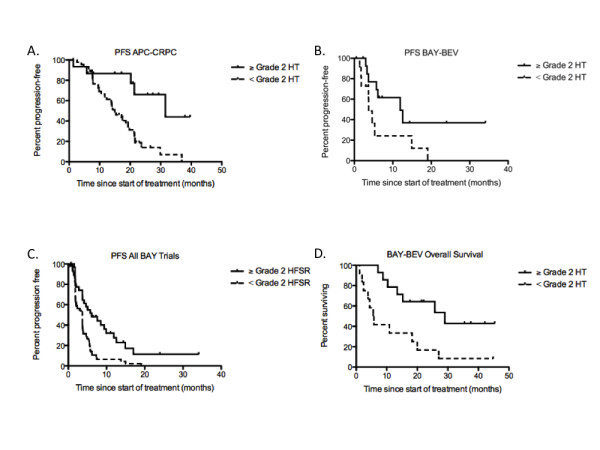
**Kaplan-Meier curve of progression-free survival following treatment with bevacizumab in combination with docetaxel and thalidomide, *n *= 60 **(A)**, or bevacizumab in combination with sorafenib, *n *= 27 **(B)**, or sorafenib alone or in combination with bevacizumab, or cetuximab in patients with prostate cancer, various solid tumors, colon cancer, or NSCLC *n *= 113 **(C)**, or overall survival following treatment with bevacizumab in combination with sorafenib, *n *= 26 **(D) **versus development of ≥ Grade 2 toxicity - - or < Grade 2 toxicity ------ as indicated on each respective figure**. Respective *P *= 0.0009, *P *= 0.052, *P *= 0.0003, and *P *= 0.0068 by a two-tailed log-rank test.

Because drug-induced toxicities may be directly related to the activity of bevacizumab and sorafenib, we hypothesized that these toxicities may also predict the progression free survival (PFS) and overall survival (OS) following anti-VEGF therapy. Patients on BAY-KS were not included in the survival analysis since this cohort was small with limited survival data. When the other 5 clinical trials presented in Table 1 were examined individually, we determined that HT was associated with prolonged PFS in patients treated with bevacizumab on the APC-CRPC and BAY-BEV trials (*P *= 0.0009, and *P *= 0.052 respectively). The median PFS difference was 14.9 (HT < grade 2, *n *= 45) versus 31.5 months (HT ≥ grade 2, *n *= 15) in patients participating on the APC-CRPC trial (Figure 1A), and 3.7 (HT < grade 2, *n *= 12) versus 11.9 months (HT ≥ grade 2, *n *= 15) for those on BAY-BEV (Figure 1B). Development of HT was not related to survival following sorafenib without bevacizumab (BAY-NSCLC and BAY-CRC; P > 0.19), with a single exception where patients on BAY-CRPC with < grade 2 HT (*n *= 37) actually had marginally non-significantly prolonged survival when compared to those individuals with HT ≥ grade 2 (*n *= 9; 1.8 versus 3.6 months respectively; *P *= 0.067).

As is indicated in Table 1, incidence of ≥ grade 2 HFSR was also associated with PFS in patients with colon cancer treated with sorafenib (*P *= 0.0065) with those patients having HFSR (*n *= 2) having a significantly longer response to sorafenib (8.7 months) than those without HFSR (4.7 months, *n *= 16). HFSR and PFS were either marginally not associated in patients on BAY-BEV (*P *= 0.094), or were not associated on BAY-NSCLC and BAY-CRPC (*P *≥ 0.29). However, since each group treated with sorafenib had a similar trend (i.e. patients with HFSR always had a longer median PFS) with a small number of patients in each group (*n *≤ 46), we pooled survival data obtained from the above trials to analyze the relationship between HFSR and PFS with greater statistical power. The pooled analysis significantly improved the relationship between PFS and HFSR with patients who developed HFSR following treatment with sorafenib, either as single agent or in combination with bevacizumab or cetuximab (*n *= 32), having a median PFS of 6.1 months compared with 3.6 months in patients without these toxicities (*n *= 81; *P *= 0.0003, Figure 1C). However, this pooled analysis should be interpreted with caution given that it is present only when heterogeneous groups of data obtained from patients are combined together. Association of these toxicities with OS was not significant with a single striking exception where those patients receiving the BAY-BEV combination had a significantly longer survival (*P *= 0.0093) if they developed hypertension during therapy (29 months, *n *= 14) when compared to those that did not develop hypertension (5.7 months, *n *= 12; Figure 1D). No other toxicity (i.e., rash/desquamation, diarrhea, or fatigue) was related to PFS (*P *> 0.05) for either drug.

### Increased risk of developing HFSR along with HT

We next hypothesized that since HT and HFSR originate from the activity of bevacizumab and sorafenib, the development of a single toxicity (i.e. HT) would increase the risk of developing the other (i.e. HFSR). Analysis of association between toxicities revealed that individuals with HT grades < 2 had a lower risk of developing HFSR grades ≥ 2 (19 of 126 patients, 15.1%) than those patients with HT grades ≥ 2 (19 of 52 patients, 36.5%, OR (95%CI) = 3.2 (1.5-6.8), *P *= 0.0024). Therefore, increased HT grade conferred a significantly increased risk of also developing HFSR.

### VEGFR2 H472Q and V297I genotypes vs. treatment associated toxicities and survival following sorafenib and/or bevacizumab therapy

The associations of HT and HFSR with the VEGFR2 H472Q polymorphism were significant when all trials were pooled (see Table 3). Frequencies of HT and HFSR for patients carrying the variant VEGFR2 H472Q polymorphism was almost double the HT/HFSR frequency of wild-type allele carriers who recieved therapies against VEGF pathway (HT: variants, 39% vs. wild-type, 21%, OR (95%CI) = 2.3 (1.2 - 4.6), *P *= 0.0154; HFSR: 33% vs. 16%, OR (95%CI) = 2.7 (1.3 - 5.6), *P *= 0.0136). Similar results were obtained for following subgroups: patients treated with only sorafenib (HT: 32% vs. 18%, *P *= 0.25; HFSR: 39% vs. 16%, *P *= 0.045) and patients treated with sorafenib as at least one of the therapies (with or without bevacizumab; HT: 42% vs. 21%, *P *= 0.0210; HFSR: 44% vs. 20%, *P *= 0.0063). These results must also be interpreted with caution given that multiple clinical trials with different toxicity incidence were pooled together. VEGFR2 genotype was not related to other toxicities (i.e., rash/desquamation, diarrhea, or fatigue; *P *> 0.05).

**Table 3:** Comparison of toxicities between wild type and variant allele groups for VEGFR2 SNPs

Toxicity grade ≥2 N (%*)	VEGFR2 H472Q			VEGFR2 V297I		
	wt allele	var allele	p-value^†^	Wt allele	var allele	p-value^†^
HT	22 (21.4)	26 (38.8)	**0.0154**	38 (29.0)	12 (30.8)	0.84
HFSR	16 (15.5)	22 (32.8)	**0.0136**	28 (21.4)	10 (25.6)	0.66
Rash:desquamation	17 (25.0)	13 (28.9)	0.67	23 (27.7)	9 (30.0)	0.82
Diarrhea	14 (20.6)	7 (15.6)	0.62	19 (22.9)	3 (10.0)	0.18
Fatigue	12 (17.7)	6 (13.3)	0.61	14 (16.9)	4 (13.3)	0.78

*% of total patients in that group,**^†^**p-values are based on Fisher's exact test. **wt**: wild-type, **var**: variant.

To determine whether the aforementioned association between HT and HFSR is confounded by VEGFR2 H472Q, the association between any two of the factors (i.e., HT, HFSR and VEGFR2 H472Q) with stratification by the remaining factor were tested. The results were consistent with the hypothesis that the associations are independent of each other. Genotype-toxicity relationships for other toxicities and studied *VEGFR2 *SNPs were not significant (Table 3). The VEGFR2 V297I SNP was not related to toxicity, and neither *VEGFR2 *genotype was related to any survival endpoint in any of the individual clinical trials in spite of the relationship with toxicity.

## Conclusions

We hypothesized that 1) increased HT and HFSR were markers for increased response duration in individuals treated with bevacizumab and/or sorafenib; 2) that since these toxicities are likely derived from the activity of bevacizumab and sorafenib, the development of HT would increase the risk of also developing HFSR; and 3) that *VEGFR2 *genotypic variation may be responsible for alterations in the activity of bevacizumab and/or sorafenib therapy that would manifest in associations with toxicity or clinical outcome following treatment with these agents. The results of the present study confirm a previously published study where HFSR development was noted to be related to PFS in patients with various solid tumors receiving doses of sorafenib between 300-600 mg bid [17], and a small study that HT is related to bevacizumab response [18]. Moreover, those receiving combination therapy with bevacizumab and sorafenib that developed hypertension enjoyed a greater than 5-fold increase in overall survival following therapy initiation. Consistent with our previous results [7], the development of HT was also directly related to the incidence of HFSR, further suggesting that these two toxicities are markers for the activity of anti-VEGF therapy. This study is the first to evaluate VEGFR2 H472Q status; carriers of 472Q alleles were more likely to experience HT and HFSR, although the relationship between genotype and toxicity was independent of the relationship between the two types of toxicity, and was not related to any of the studied survival endpoints.

The physiological basis for bevacizumab- and sorafenib-induced HT and HFSR is currently unknown although they most likely originate from the activity of these drugs altering signaling through several targets (i.e., VEGF, Raf-1, wild-type B-Raf, mutant b-raf V599E, VEGFR2, VEGFR3, PDGFR-β, Flt3, c-KIT and p38) [19,20]; recent data suggests that the VEGF pathway directly contributes [6,7]. Once these pathways are altered, HT may develop because of decrease in vascular surface area [6], and HFSR may develop due to inefficiency of the repair of microtrauma originating from use of the hands and feet [21]. In spite of the unknown origin of these toxicities, our data are consistent with the hypothesis that HT and HFSR are related to the activity of these drugs. The data also suggest that these toxicities are markers for prolonged response, and in the case of sorafenib and bevacizumab coadministration, prolonged survival benefit from these therapies. Others have also observed that the severity of rash in patients with NSCLC is directly related to EGF-RTK inhibition by tyrosine kinase inhibitors, and that this cutaneous toxicity is also a marker for increased survival [17,22]. Moreover, it has also been suggested that rash brought on by EGF-pathway inhibitors could be useful for optimal dose titration [17]. Therefore, future studies directly evaluating the development of HT and HFSR as markers for effective dosing of these bevacizumab and sorafenib are warranted in order to decrease the incidence of toxicity and improve response.

Interestingly, *VEGFR2 *genotype may also be related to the incidence of both HT and HFSR independently, but does not confound the relationship between the two toxicities. These data suggest that the development of these toxicities is related to signaling through the VEGF pathway, at least in part, although the polymorphism in *VEGFR2 *is not the sole factor responsible for the relationship between HT and HFSR. Given the heterogeneity of the clinical trials under study, the lack of a relationship between *VEGFR2 *genotype and PFS may be due to low statistical power and it is hoped that future studies in homogeneous populations will validate the relationship between *VEGFR2 *polymorphism and survival.

The present analysis is inconsistent with a previous report where it was determined that patients with breast cancer reported significantly longer OS for patients who developed HT on bevacizumab and paclitaxel combination than patients without this toxicity [23]. The present data were obtained retrospectively from clinical studies that were not designed to retain patients on the basis that toxicity was a marker for efficacy. Indeed, a greater proportion of patients carrying the 472H/Q substitutions were removed from the trials due to toxicity (14%) than those carrying wild-type or variant genotypes (9%), although this was not statistically significant (data not shown). This is not surprising given the association of *VEGFR2 *variants and toxicity. However, since those carrying this genotype also had a better response in general, it is possible that the desirable long-term benefit of the treatment may not have been enjoyed in patients being removed from therapy prior to tumor progression due to toxicity.

In conclusion, our data indicate that HT and HFSR are markers for prolonged progression free survival in patients treated with bevacizumab and/or sorafenib, patients receiving a combination of both agents that develop HT have a large increase in treatment-related survival, and that the development of HT on these agents increases the risk of also developing HFSR. The association with toxicity was not significant with respect to overall survival. When *VEGFR2 *genotypes were considered, the present data suggest that those carrying 472Q alleles at H472Q are at an increased risk of developing both HT and HFSR following bevacizumab, although the SNP is not related to either progression free survival or overall survival. Given the exploratory pilot nature of this study, it is hoped that future studies will validate these results and provide a mechanism by which toxicity is related to PFS and *VEGFR2 *genotypic variation is related to toxicity.

## Competing interests

The authors declare that they have no competing interests.

## Authors' contributions

LJ, TMS, BCE, CEB, and DKP carried out experiments; ECK, WLD, SK, RY, and GG treated the patients and collected the data for the study; LJ, TMS, DV, and DL conducted final statistical analysis; Study was conceived by TMS, RD, JV, and WDF; WDF provided financial support. All authors have read and approved the final manuscript.
